# Habitat-mediated breeding performance of Lewis’s Woodpeckers (*Melanerpes lewis*) in British Columbia

**DOI:** 10.1371/journal.pone.0212929

**Published:** 2019-03-20

**Authors:** Lauren Macfarland, Nancy A. Mahony, Megan Harrison, David Green

**Affiliations:** 1 Simon Fraser University, Centre for Wildlife Ecology, Department of Biological Sciences, Burnaby, British Columbia, Canada; 2 Environment and Climate Change Canada, Wildlife and Research Division, Science and Technology Branch, Edmonton, Alberta, Canada; 3 Environment and Climate Change Canada, Canadian Wildlife Service, Delta, British Columbia, Canada; Institute of Systematics and Evolution of Animals Polish Academy of Sciences, POLAND

## Abstract

Tree cavities provide a critical resource for cavity-nesting animals, and high quality cavities can be difficult for animals to acquire in habitats where competition is high. We investigated the breeding performance of Lewis’s Woodpeckers in three habitat types in British Columbia, Canada in 2013 and 2014. We also assessed whether the number of nest competitors and cavity availability influenced the habitat specific breeding performance of this threatened cavity nesting species. We found that daily nest survival rate was lower in burned habitat (0.15 ± 0.08 (0.05–0.37)) than in live pine (0.72 ± 0.10 (0.51–0.87)) or cottonwood (0.69 ± 0.09 (0.51–0.83)) habitats. However, hatching success (the proportion of eggs that hatch) was lower in live pine habitat (0.59 ± 0.09 95% CI) than burned (0.77 ± 0.19 95% CI) or cottonwood (0.80 ± 0.07 95% CI) habitat, and the fledging success of successful nests in live pine and burned habitat (1.86 ± 0.31 and 1.88 ± 0.59 95% CI, respectively) was slightly lower than in cottonwood habitat (2.61 ± 0.45 95% CI). Consequently, Lewis’s Woodpeckers in cottonwood habitat produced more fledglings per nesting attempt (2.05 ± 0.49 95% CI) than in live pine (1.53 ± 0.35 95% CI) or burned (0.79 ± 0.49 95% CI) habitat. Habitats differed in the number of nesting competitors and the number of suitable cavities surrounding active Lewis’s Woodpecker nests. Our results showed that cavity density best explained breeding performance differences although the mechanisms remain unclear. There was no evidence that the number of heterospecific nest competitors, including the invasive European Starling (*Sturnus vulgaris*), explained or influenced Lewis’s Woodpecker breeding performance. Cavity density influenced the productivity of successful nests but did not explain habitat differences in hatching success or daily nest survival. Further work is required to understand the mechanistic basis for the habitat specific breeding performance of Lewis’s Woodpeckers. Habitat differences in breeding performance in British Columbia are not consistent with those in other regions, highlighting the importance of regionally-specific demographic data for managing species at risk.

## Introduction

Tree cavities provide critical breeding habitat for a large number of vertebrates and individuals may increase their fitness by choosing cavities that lead to high reproductive success [1–4]. The quality of cavities affects the reproductive success of many cavity-nesting species because cavity size and entrance height, shape and orientation can all influence the impact of extreme weather events and predation [5–10]. Consequently, cavity availability can be a limiting factor for some populations of mammals [11], birds [12], and amphibians [13]. Identifying habitats that support higher densities of high quality cavities, that produce higher breeding success, may therefore be important for supporting populations of cavity-nesters of conservation concern.

Tree cavities may be formed naturally by rot fungi and bacteria, or by excavating animals (primarily woodpeckers) [14, 15]. In northern temperate forests, the community of cavity-nesting species has been described as a “nest web” [14]. The nest web is composed of primary cavity-nesters, which are strong excavators that create the majority of cavities, weak cavity-nesters that are less adapted for excavation and rarely do so, and secondary cavity-nesters that do not excavate, but rather use already formed cavities, either excavated by primary and weak cavity-nesting species, or formed by the naturally occurring decay process.

The extent to which competition for high quality nest cavities regulates populations of weak and secondary cavity-nesting birds may vary across habitat types depending on the abundance of primary cavity-nesters, the abundance of competitor species, and outcomes of competitive interactions between other species in the nest web. Competition for high quality nest sites may be less important in habitats with abundant dead or decaying trees, which provide the raw material for the creation of cavity nests, or when primary cavity-nesters are abundant [16]. Competition may be more important when weak and secondary cavity-nesters are forced to interact with interspecific competitors, like the European Starling (*Sturnus vulgaris*), that aggressively compete for nesting cavities [17, 18].

Lewis’s Woodpeckers (*Melanerpes lewis*) are weak cavity-nesting birds whose populations are declining across their range in western North America (-3.42% per year between 1966 and 2015) [19]. In 2012, Lewis’s Woodpeckers were listed as a Threatened species in Canada [20–22]. In British Columbia, Lewis’s Woodpeckers currently only occupy areas within the southern interior of the province where they nest in three distinct habitat types: riparian black cottonwood (*Populus trichocarpa*), live ponderosa pine (*Pinus ponderosa*) and crown-burned mixed coniferous habitats (hereafter cottonwood, live pine and burned habitat). This region has experienced significant loss and degradation of these three forest types since pre-settlement times [21]. Cottonwood and live pine habitat has been transformed by dam and reservoir development, livestock grazing or conversion of habitat to agriculture or urban use. In the Okanagan Valley, the center of Lewis’s Woodpecker range in British Columbia, cottonwood habitat occupies only a fraction of its original area (up to 86% lost) and live pine habitat has decreased by 50% [23]. Fire regimes have also changed drastically since historic times. Both high and low-intensity fires that once occurred frequently, promoting an open understory and regeneration of native vegetation, have become less frequent through active fire suppression [24], potentially reducing the amount of available habitat. Populations of non-native European Starlings, which expanded into British Columbia in the 1960s, could also have contributed to population declines of Lewis’s Woodpeckers through aggressive competition for nesting sites [20, 21].

Although Lewis’s Woodpeckers are considered “burn specialists” [25, 26], nest success and productivity of Lewis’s Woodpeckers varies among habitat types throughout their range. For instance, Lewis’s Woodpeckers in burned habitat in Idaho and Wyoming have high nest success and productivity [25, 27] while burned habitat in an earlier study on and near our study sites in British Columbia was found to have relatively low nest success and productivity [7]. In another instance, Lewis’s Woodpeckers in cottonwood habitat in Montana have high nest success and productivity [28] while those in Colorado have relatively low nest success and productivity [25]. Saab and Vierling [25] suggest that there may be differences in predators or food availability across habitat types that cause differences in breeding performance. However, the mechanisms driving variation in nest success and productivity remain unclear.

In this study we aim to 1) examine differences in the reproductive success of Lewis’s Woodpeckers across three habitat types in British Columbia, Canada, 2) compare these reproductive results to previous studies within and outside this region, 3) examine differences in abundance of cavity availability and native and non-native nest competitors across habitats, and 4) assess whether competition for cavities limit Lewis’s Woodpecker breeding performance.

## Methods

### Study area

Our study was located within the core of the Lewis’s Woodpecker’s Canadian range, the south Okanagan Valley and Boundary area of interior British Columbia. Within this larger area we grouped multiple nests found within 1.5 km of each other (the maximum distance we observed individuals traveling from the nest tree to forage) into sites. All sites had the characteristically open tree canopy (~30% cover) and fairly dense understory (~60% cover) that Lewis’s Woodpeckers prefer, which creates ample space for flycatching and sufficient shrub cover for harboring insects for forage [29].

Sites were located in three distinct habitat types. Live ponderosa pine sites were between 350 and 675 m in elevation and located 10 km west of Osoyoos (49°05’N, 119°36’W) and near Oliver, British Columbia (49°18’N, 119°31’W). Tree composition included ponderosa pine exclusively, and the understory vegetation was mainly antelope-brush (*Purshia tridentate*) and sagebrush (*Artemisia tridentate*). The burned sites were between 400 and 1100 m in elevation, located near the town of Okanagan Falls, BC east of Vaseux Lake (49°17’N, 119°31’W) and 3 km east of Osoyoos, British Columbia (49°01’N, 119°23’W). Tree composition included Douglas fir (*Pseudotsuga menziesii*) and ponderosa pine that burned at high-intensity 10–15 years ago. The most frequent understory shrubs were snowberry (*Symphoricarpos albus*), wax currant (*Ribes cereum*), and Oregon grape (*Mahonia aquifolium*). Cottonwood sites were between 500 and 600 m in elevation, located within the towns of Rock Creek (49°03’N, 119.00’W), Midway (49°00’N, 118°49’W), and Grand Forks, British Columbia (49°01’N, 118°27’W). These sites were composed of narrow strips (5 to 20 m wide) of forest patches that paralleled the Kettle and Granby Rivers. Open areas containing agricultural, industrial or residential property were often adjacent to these cottonwood sites. Tree composition included primarily black cottonwood and secondarily, Douglas fir, although a few small stands of water birch (*Betula occidentalis)* and trembling aspen (*Populus tremuloides*) were present. Understory vegetation included Oregon grape, snowberry and saskatoon (*Amelanchier alnifolia*).

### Field methods

During 2013 and 2014, we located active Lewis’s Woodpecker nests by searching areas where previous nesting attempts had been documented by the Canadian Wildlife Service [22] and Zhu et al. [7]. We found all nests during the pre-laying, nest renovation or egg-laying stages. We visited nests at intervals of 2 to 5 days, following Dudley and Saab’s guidelines for monitoring cavity-nesting birds [30]. We were able to check contents of nests in cavities up to 16 m in height using wireless telescoping cameras, the TreeTop Peeper [Sandpiper Technologies, Maneca, California] and the Peeper Cam [IBWO.org David Luneau, Arkansas]. We recorded the final clutch size, hatching success (the proportion of eggs that hatched), brood size seven days post-hatch, fledging success (the proportion of young that fledged from the brood), and the productivity measured as the number of fledglings last observed in the nest cavity before the expected date of fledging. We confirmed the number that fledged by visiting nests multiple times for the best possible chance of detecting fledgling activity. For nests that we were unable to reach with equipment, we used parental behaviors to indicate the nesting stage. For example, an adult carrying food into the cavity showed that eggs had hatched and nestlings were present. We were unable to determine the clutch sizes, hatching success or fledging success for these nests, but we determined the minimum productivity by counting recently fledged young on or very near the nest cavity. We are confident in using the number of recently fledged young as a measure of the true number of fledglings because for nests with known brood size, we detected all fledglings observed in the cavity just prior to fledging.

To quantify the level of potential nest competition, we recorded nest locations of European Starlings, American Kestrels, Northern Flickers (*Colaptes auratus)*, and other Lewis’s Woodpeckers within 50 m (0.785 ha) of each monitored Lewis’s Woodpecker nest. These four species occupy cavities of similar sizes [2, 31], and are considered competitors for the same nest cavities. Within the same area we also counted the number of surplus cavities, the number of cavities unused by any species but suitable for use by Lewis’s Woodpeckers. We determined whether cavities could be considered “surplus” (unused) by observing that cavities were not used during May and June of each year. We considered a cavity to be suitable if it appeared fully excavated with an entrance diameter between 5 and 10cm.

We conducted this research ethically and in compliance with Animal Care permits issued by the Canadian Council of Animal Care (permit no. 1081B-13).

### Data analysis

We modelled daily nest survival rates using the Program MARK version 8.0 [32, 33], and calculated the cumulative expected survival for a six-egg clutch, 14 day incubation period and 30 day nestling period [34]. We first examined habitat effects on daily nest survival in a candidate set that included a null model and all combinations of habitat, season (day of year, where January 1 = 1), and period (laying/incubation or nestling; n = 8 models). Next, we asked if habitat effects on daily nest survival rates could be explained or improved by the addition of covariates describing the number of heterospecific and conspecific competitors, cavity density, and surplus cavities within 50 m (0.785 ha) of the nest. This candidate set also included eight models. We used Akaike’s Information Criterion corrected for small sample sizes (AICc; [35]) and Akaike weights (wi) to rank and compare models in each candidate set.

We then performed three modeling efforts to assess the influence habitat type, competitors, cavity density, and surplus cavities on Lewis’s Woodpecker 1) nest survival, 2) hatching success, and 3) productivity. We then used general linear mixed models, that included site as a random factor, to compare the hatching success and productivity using Lewis’s Woodpecker nests for which we were able to collect data in 2013 and 2014. We treated hatching success as binomially distributed, and productivity (fledgling number for successful nests) as poisson-distributed. Each of the two candidate model sets allowed breeding performance to vary depending on the habitat type, number of heterospecific and conspecific competitors, cavity density, and surplus cavities. We also included a null model. Therefore, each candidate model set included ten models (Table 1).

**Table 1:** Summary of candidate model sets examining how habitat, cavity density, competitors and surplus cavities influences the breeding performance of Lewis’s Woodpeckers in British Columbia, Canada.

Model	Variables	Justification
Null	Null	
Habitat	Habitat	Breeding performance of Lewis’s Woodpeckers can vary across habitat types [25].
Cavity Density	Cavity Density	Cavities are a limited resource [12]. Fewer cavities may mean less choice in a nesting cavity, possibly forcing Lewis’s Woodpeckers to use low quality cavities.
Competitors	Competitors	Heterospecific competitors may have negative direct (cavity usurpation) and indirect (reduction of cavity availability) effects on Lewis’s Woodpecker breeding performance.
Lewis’s Woodpeckers	Lewis’s Woodpeckers	Benefits of group living may include locating high quality foraging areas through social learning or group nest defense against predators [36].
Surplus Cavities	Surplus Cavities	Cavities are a limited resource [12]. Lewis’s Woodpeckers may be forced to use low quality cavities when experiencing high competition.
Habitat and Cavity Density	Habitat + Cavity Density	Habitat effects are not solely explained by habitat differences in cavity density. More cavities nevertheless means there is more choice in a higher quality cavity.
Habitat and Competitors	Habitat + Competitors	Habitat effects are not solely explained by habitat differences in nesting heterospecific competitors surrounding Lewis’s Woodpecker nests. However, more nesting competitors may have negative effects on Lewis’s Woodpecker breeding performance because less high quality cavities would be available to use.
Habitat and Lewis’s Woodpeckers	Habitat + Lewis’s Woodpeckers	Habitat effects are not solely explained by habitat differences in nesting conspecific competitors. However, there may be positive effects of group living [36].
Habitat and Surplus Cavities	Habitat + Surplus Cavities	Habitat effects are not solely explained by the number of surplus cavities. More surplus cavities nevertheless could indicate more choice to obtain higher quality cavities.

We used Akaike’s Information Criteria adjusted for small sample sizes (AICc) to calculate model weights for each model [37]. We chose the best models by using AICc model weights that quantify the strength of support for each model relative to the other included models. We used program R v3.1.3 [38] for all analyses, and called package ‘lme4’ to build generalized linear mixed effects models and package ‘MuMIn’ to calculate model-averages.

## Results

### Habitat effects

In total, we recorded 95 nest attempts by Lewis’s Woodpeckers in 2013 and 2014 across cottonwood, live pine and burned habitat types (Table 2). We monitored nests at 21 sites: ten in live pine, four in burned, and seven in cottonwood habitat. Lewis’s Woodpeckers laid clutches containing 5.94 ± 2.46 SD eggs (range = 3–11, n = 54). We are confident that the clutch sizes we recorded were complete because only one nest failed during the laying period (when it contained four eggs). We observed two nests with three-egg clutches and both successfully fledged young. We were able to access 54 nests with equipment. Nests in live pine habitat were more easily accessible than nests in cottonwood and burned habitat (77% nests accessed in live pine, 47% in cottonwood, and 42% in burned). Clutch size was independent of habitat type. However, hatching success varied across habitats and, consequently, pairs nesting in cottonwood habitat had significantly larger broods seven days post-hatch compared to those nesting in burned and live pine habitat (Table 3). Fledging success did not vary significantly with habitat type, although the productivity per successful nest was higher in cottonwood habitat than in burned or live pine habitat (Table 3). Over the two years of this study, Lewis’s Woodpeckers had far higher nest survival in cottonwood and live pine habitat than in burned habitat. However, Lewis’s Woodpeckers nesting in cottonwood habitat were more productive than pairs in both live pine and burned habitat (Table 3).

**Table 2:** Total number of Lewis’s Woodpecker nests monitored in both years and the extent of nest monitoring overlap in 2014 across three habitat types.

	Cottonwood	Live Pine	Burned
Total No. Nests Monitored	42	34	19
No. Nests Monitored 2013	21	18	8
No. Nests Monitored 2014	21	16	11
No. Same Nest Cavities In Both Years (%)	6 (14%)	9 (26%)	3 (16%)

**Table 3:** Habitat-specific breeding performance, including daily nest survival rates (mean ± 95% CI), of Lewis’s Woodpeckers in British Columbia, Canada.

	Cottonwood	Live Pine	Burned	Statistic	Pvalue
Clutch Size_a_	6.30 ± 0.78 (n = 20)	5.69 ± 1.52 (n = 26)	5.88 ± 1.35 (n = 8)	F = 1.22	0.30
Hatching Success_b_	0.80 ± 0.07 (n = 18)	0.58 ± 0.08 (n = 26)	0.77 ± 0.19 (n = 7)	F = 4.64	0.01
Brood Size_c_	5.11 ± 0.69 (n = 18)	3.35 ± 0.65 (n = 26)	4.71 ± 1.80 (n = 7)	F = 5.98	0.004
Daily Nest Survival (Egg Laying)	0.998±0.001 (0.993–0.999) (n = 95)	0.998±0.001 (0.993–0.999) (n = 95)	0.987±0.006 (0.969–0.995) (n = 95)	N/A	N/A
Daily Nest Survival (Nestling Period)	0.993±0.002 (0.993–996) (n = 95)	0.994±0.003 (0.986–0.9978) (n = 95)	0.965±0.010 (0.938–0.980) (n = 95)	N/A	N/A
Daily Nest Survival (Cumulative)	0.69±0.09 (0.51–0.83) (n = 95)	0.72± 0.10 (0.51–0.87) (n = 95)	0.15±0.08 (0.05–0.37) (n = 95)	N/A	N/A
Productivity per Successful Nest_d_	2.61 ± 0.45 (n = 33)	1.86 ± 0.31 (n = 28)	1.88 ± 0.59 (n = 8)	F = 0.65	0.52
Productivity per Attempted Nest_e_	2.05 ± 0.49 (n = 42)	1.53 ± 0.35 (n = 34)	0.79 ± 0.49 (n = 19)	F = 3.9	0.02

a determined after no additional eggs were laid after two consecutive nest visits

b proportion of eggs in a complete clutch that hatched

c number of nestlings observed seven days post-hatch

d number of fledglings recorded near the nest during the fledging period for nests able to fledge at least one young

e number of fledglings recorded at each nest, including those nests that failed to produce any young

There was considerable variation in the number of suitable cavities, nest competitors, and surplus cavities within 50 m of nests across the three habitat types (See S1 Table). Cavities were most abundant in cottonwood habitat and least abundant in live pine habitat, where live trees may inhibit the excavation of cavities. The number of nesting Northern Flickers and American Kestrels did not differ across habitat types. However, European Starling nests were most abundant in cottonwood habitat and lowest in live pine. There were more than two times the number of heterospecific competitors in cottonwood habitat than burned and live pine habitat. Conspecifics were also more abundant within a 50 m radius of Lewis’s Woodpecker nests in cottonwood and burned habitat types when compared to the live pine habitat type that had no nesting Lewis’s Woodpeckers within 50 m of any nest (S1 Table). Despite the greater number of competitors, there were significantly more surplus cavities in cottonwood habitat than in both burned and live pine habitat types (S1 Table).

### Factors influencing habitat-dependent breeding performance

Daily nest survival rates were lower during the nestling period than the laying/incubation period and lower at nests in burned habitat than cottonwood or live pine habitat (Table 4). The top model in the first candidate set examining daily nest survival rates included the habitat and nesting period variables. This model received more than 2-times the support of the more complex model that also allowed daily survival rates to vary seasonally, and 100-times the support of the null model (Table 4). Cumulative expected nest survival rates were estimated to be >4-times higher in cottonwood and live pine habitat than in burned habitat (Table 3). Habitat effects on daily nest survival rates were not explained by differences in the number of heterospecific and conspecific competitors, cavity density or the number of surplus cavities. The top model in the second candidate set remained the best model in the first candidate set, i.e. the model with the habitat and period variables (Table 4).

**Table 4:** Daily nest survival model candidate sets for Lewis’s Woodpecker showing relative support for a) habitat, season and nesting period (laying/incubating vs. nestling) effects, and b) habitat, nestlings, cavity and competitor effects.

**a) Daily Nest Survival with habitat, season and nesting period effects (n = 95)**	K	ΔAICc	w_i_
Habitat + Nesting Period	4	0.00	0.55
Habitat + Season + Nesting Period	5	1.67	0.24
Habitat	3	3.14	0.11
Habitat + Season	4	3.51	0.09
Nesting Period	2	13.21	0.00
Null	3	14.89	0.00
Season + Nesting Period	3	15.12	0.00
Season	2	15.39	0.00
**b) Daily Nest Survival with covariate effects (n = 95)**	K	ΔAICc	w_i_
Habitat + Nesting Period	4	0.00	0.32
Habitat + Nesting Period + Cavity Density	5	0.95	0.20
Habitat + Nesting Period + Lewis’s Woodpeckers	5	1.05	0.19
Habitat + Nesting Period + Surplus Cavities	5	1.51	0.15
Habitat + Nesting Period + Heterospecific Competitors	5	1.85	0.13
Nestling + Lewis’ Woodpeckers	3	13.78	0.00
Nestling + Cavity Density	3	14.13	0.00
Nestling + Surplus Cavities	3	14.48	0.00
Nestling + Heterospecific Competitors	3	15.21	0.00

The top model in the candidate set examining the influence of cavity availability and competitors on hatching success indicated that the proportion of eggs that hatched varied with habitat type (Table 5), confirming the previous analysis (Table 3). Models with either the Surplus Cavity or Cavity Density term in addition to the Habitat term received similar support to the top model. However, parameter estimates for the Surplus Cavity and Cavity Density terms had confidence intervals that bounded zero suggesting that cavity effects were weak.

**Table 5:** Summary of complete candidate model sets examining the influence of habitat, cavity density, competitors, and surplus cavities on the breeding performance of Lewis’s Woodpeckers in British Columbia, Canada. The two analyses included evaluation of Hatching Success (the proportion of eggs that hatch), and Productivity (number of fledglings produced by each successful nest).

**Hatching Success (n = 51)**	K	ΔAICc	w_i_
Habitat	4	0.00	0.25
Habitat + Surplus Cavities	5	0.23	0.23
Habitat + Cavity Density	5	0.71	0.18
Habitat + Heterospecific Competitors	5	1.83	0.10
Habitat + Lewis’s Woodpeckers	5	2.09	0.09
Surplus Cavities	3	2.71	0.07
Cavity Density	3	2.89	0.06
Lewis’s Woodpeckers	3	5.55	0.02
Null	2	7.81	0.01
Heterospecific Competitors	3	9.96	0.00
**Productivity of Successful Nests (n = 67)**	K	ΔAICc	w_i_
Cavity Density	3	0.00	0.25
Surplus Cavities	3	0.32	0.22
Null	2	1.41	0.12
Habitat	4	1.62	0.11
Lewis’s Woodpeckers	3	2.82	0.06
Habitat + Surplus Cavities	5	2.90	0.06
Habitat + Cavity Density	5	3.17	0.05
Habitat + Heterospecific Competitors	5	3.48	0.04
Heterospecific Competitors	3	3.57	0.04
Habitat + Lewis’s Woodpeckers	5	3.95	0.03

**Fig 1:**
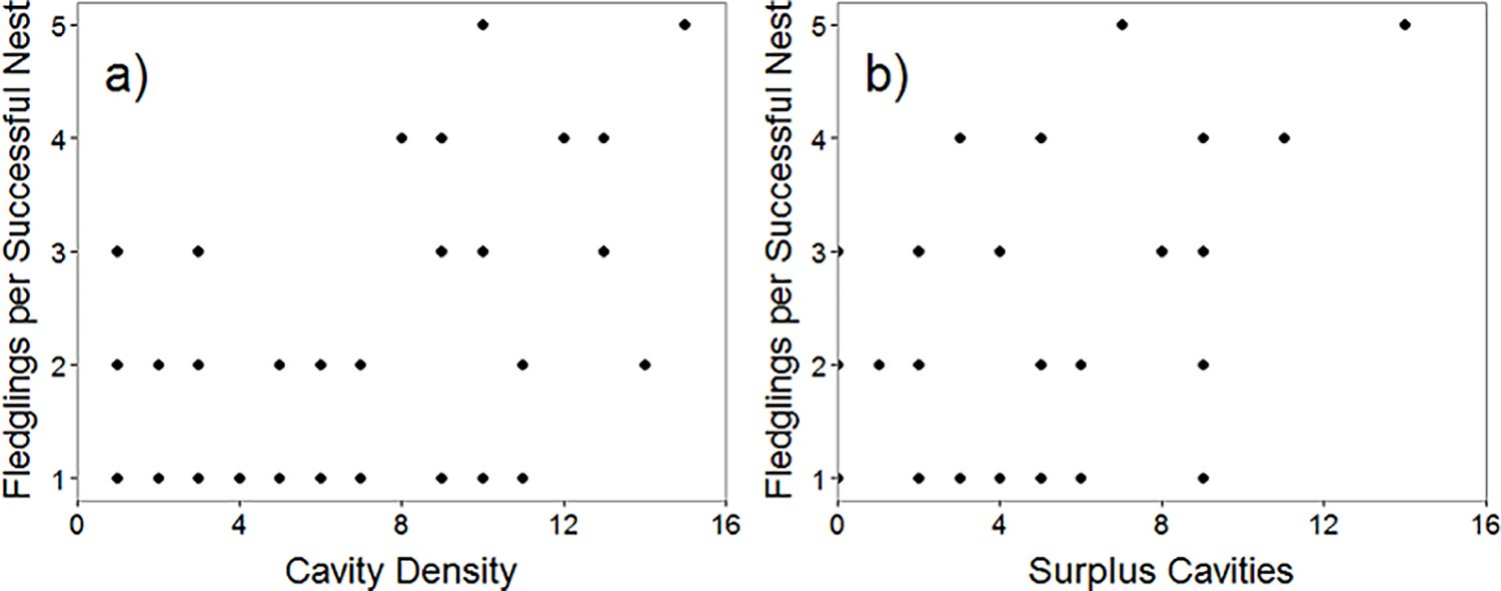
**Relationship between a) cavity density and fledging success (r = 0.31, p<0.01) and b) surplus cavities and fledging success (r = 0.30, p<0.01) of Lewis’s Woodpeckers in British Columbia, Canada in 2013 and 2014. Fledging success is the number of fledglings produced per successful nest.** The top two models in our AICc analysis investigating the effects of cavity availability and competitors on Lewis’s Woodpecker breeding performance suggest that cavity density and surplus cavities have the greatest influence on productivity (number of fledglings) of successful nests.

The two top models in the candidate set examining variation in the productivity of successful nests included only the Cavity Density or Surplus Cavities term. These models had twice the support of the null model, and suggested that the number of fledglings produced was lower at nests where cavity density or the number of surplus cavities was low (Table 5, Fig 1). However, the habitat model received less support than the null model, confirming the results of previous analysis that found no significant habitat effects on productivity of successful nests (Table 3).

## Discussion

Lewis’s Woodpeckers are weak cavity-nesters, prone to using tree cavities created by primary cavity-nesters in cottonwood, live pine and burned habitat within British Columbia, Canada. We found that hatching success and brood size was higher for nests in cottonwood and burned habitat compared to live pine habitat. Daily nest survival was higher for nests in cottonwood and live pine habitat compared to burned habitat, and therefore overall productivity was higher for nests in cottonwood and live pine habitat compared to burned habitat. Predicted cumulative nest survival rates and nest success are consistent with previous studies on Lewis’s Woodpeckers in Montana [28] and British Columbia [7], but not consistent with studies on Lewis’s Woodpeckers in Colorado, South Dakota, and Idaho [27, 25] (Table 6). A study in Montana found that nest success and productivity of Lewis’s Woodpeckers within cottonwood habitat was high and comparable to our results from cottonwood habitat in British Columbia [28]. We monitored Lewis’s Woodpeckers at some of the same sites in British Columbia as Zhu et al. [7], who combined data from live pine and burned habitat, and found that nest success and productivity was relatively low and comparable to the combined results from live pine and burned habitat from our study (Table 6). Breeding performance of Lewis’s Woodpeckers in British Columbia may have previously been underestimated in Zhu et al.’s [7] study because they monitored fewer nests in the more productive cottonwood habitat compared to this study. In contrast to our results, a study in Colorado [25] found that Lewis’s Woodpeckers within cottonwood habitat in Colorado had much lower nest success and productivity than those within cottonwood habitat in our study (Table 6). This could be due to higher grazing pressure within cottonwood habitat in Colorado, which has diminished the understory vegetation, likely reducing food availability for woodpecker nest provisioning [25]. A study in South Dakota [27] found that nests within burned habitat (17–20 years post-fire) had high nest success and productivity, and a study in Idaho [25] found that Lewis’s Woodpeckers in burned habitat (2–7 years post-fire) had high nest success but moderate productivity. Both of these findings are in contrast to our results of low nest success and low productivity in burned habitat (10–15 years post-fire) in British Columbia (Table 6). It is not likely that differences across regions in nest success and productivity within burned sites can be explained by differences in fire history because this study and the study from South Dakota [27], which both took place within “older burns” (forests burned at a high intensity between 10–20 years prior) had opposite results. These regional differences in breeding performance mean that conservation planning for this species of concern may require recommendations that target regionally important factors affecting productivity.

**Table 6:** Regional variation in nest success (the production of ≥1 fledgling) and productivity per successful nest (number of fledglings) in Burn, Cottonwood and Live Pine habitat types. Zhu et al. [7] productivity results reflect the productivity of all attempted nests.

Habitat Type	Location	Year	Nest Success	Productivity	Source
Burn	British Columbia	2015	42% (n = 19)	1.80 (n = 8)	This study
Burn	South Dakota	2007	90% (n = 55)	3.42 (n = 50)	[27]
Burn	Idaho	2001	78% (n = 283)	1.78 (n = 221)	[25]
Cottonwood	British Columbia	2015	79% (n = 42)	2.60 (n = 33)	This study
Cottonwood	Montana	2013	89% (n = 18)	3.06 (n = 16)	[28]
Cottonwood	Colorado	2001	46% (n = 65)	1.70 (n = 30)	[25]
Live Pine	British Columbia	2015	80% (n = 36)	1.86 (n = 29)	This study
Live Pine and Burn	British Columbia	2015	67% (n = 55)	1.84 (n = 37)	This study
Live Pine and Burn	British Columbia	2012	52% (n = 43)	1.78 (n = 43)	[7]

Competition for limited high quality cavities with European Starlings and native cavity-nesting birds has been argued to be a potential threat to Lewis’s Woodpecker populations [39]. European Starlings are an invasive species known to be particularly aggressive secondary cavity-nesters that can outcompete other cavity-nesting birds for nest sites [39, 40]. However, a comprehensive study on the European Starling effect on cavity-nesting birds failed to find evidence for this hypothesis [41]. Although we typically found multiple nests of heterospecific competitors near Lewis’s Woodpecker nests in this study, we found little evidence that competitors, including European Starlings, had large effects on habitat-based variation in breeding performance of Lewis’s Woodpeckers. The competitor model that included the number of competitors surrounding each Lewis’s Woodpecker nest was not supported in any of the three model sets investigating the influences on breeding performance between the three habitat types. Although there was variation in the number of surplus holes across habitat types, it is possible that competition is minimal because the abundance of nesting competitors was sufficiently low. Over the past 50 years, Breeding Bird Surveys show steady declines of European Starling populations in British Columbia (-3.7% per year, [19]). Starling populations may have been substantially reduced across the 10-year period between Zhu et al.’s study [7] and this one, and may have declined sufficiently to no longer be a threat to Lewis’s Woodpeckers. Although we cannot conclude that there is an effect of nesting competitors (native or non-native) on Lewis’s Woodpecker breeding performance, we recommend that conservation measures include the retention of standing dead trees when possible because the loss of cavity trees may create more competition for nesting holes.

Shortages of nest cavities are well documented to limit populations of some secondary and weak cavity-nesting birds [12, 42]. In this study, cavity density did not explain habitat differences in daily nest survival rates. Cavity density did influence the productivity of successful nests, and since cavity density was significantly higher in cottonwood habitat than other habitats, it appears to contribute to habitat differences in overall productivity of Lewis’s Woodpeckers in British Columbia.

In this study, we investigated possible nest competitor and cavity availability effects on habitat-based breeding performance of Lewis’s Woodpeckers in British Columbia. However, there are other plausible mechanisms that we were unable to explore. For example, variation in the landscape matrix that may affect food availability or quality across habitat types may affect productivity [40], but was not measured. Many of our sites within cottonwood habitat appeared to be in close proximity to agricultural crops and urban areas, which may allow for a relative increase in the availability and diversity of food, such as insects, fruit crops, and backyard feeders. A second alternative explanation is that habitat types differ in the amount of exposure that nests have to predators. It is possible that sites 10–20 years post-burn (like ours) present increased foraging opportunities for generalist nest predators. If burned sites are targeted foraging areas of nest predators, then nests in burned habitat may have a greater chance of being opportunistically depredated. A longer study may further elucidate the complex ecological and environmental influences on annual variation in daily nest survival and productivity. It may also encourage further research into variation in food quality and differences in nest exposure to predators as potential drivers of habitat-based breeding performance in Lewis’s Woodpeckers.

## Conclusion

Here we show that cottonwood habitat may provide critical habitat for Lewis’s Woodpeckers in British Columbia, because hatching success, daily nest survival, and productivity of Lewis’s Woodpeckers is high in this habitat type. In British Columbia, cottonwood habitat comprises less than 20% of the designated critical habitat, which includes all three habitat types [43]. Therefore, management for high quality nesting habitat must consider both the protection of spatially limited strips of riparian vegetation along river valleys and protection of the more widespread, but potentially lower quality live pine and burned areas. Previous work has suggested that the Lewis’s Woodpecker is a burn-specialist species. However, burned forests do not appear to provide the most productive habitat in this region. Although non-native European Starlings are suspected to play a role in the decline of Lewis’s Woodpecker populations, our data suggest they do not influence breeding performance differences among habitats in British Columbia. Our results instead suggest that the low productivity particularly in burned habitat may be due, in part, to limitation of nesting cavities. We suggest that other factors such as the quality of food provisioned to nestlings or variation in nest exposure to predators should be examined. We highlight that our results differ from those in some other regions, suggesting that conclusions regarding critical habitat for this species at risk should use region-specific breeding performance data.

## Supporting information

S1 Table Variation (mean ± 95% CI) in cavity abundance, competitors, and surplus cavities within 50 m (0.785 ha) of Lewis’s Woodpecker nests across three habitat types in British Columbia, Canada. (DOCX)
