# Supplementary material for: Habitat-mediated breeding performance of Lewis’s Woodpeckers (Melanerpes lewis) in British Columbia
Source: PLoS One. 2019 Mar 20;14(3):e0212929. doi: 10.1371/journal.pone.0212929 (PMC6426180; doi:10.1371/journal.pone.0212929)
Supplement: S1 Table — (DOCX) [file pone.0212929.s001.docx]

**Supporting Information**

**S1 Table. Variation (mean ± 95% CI) in cavity abundance, competitors, and surplus cavities within 50 m (0.785 ha) of Lewis’s Woodpecker nests across three habitat types in British Columbia, Canada.**

|  | **Cottonwood** | **Live Pine** | **Burned** | **Statistic** | **Pvalue** |
| --- | --- | --- | --- | --- | --- |
| Number of Cavities | 9.28 ± 1.63 (n=40) | 3.63 ± 1.02 (n=34) | 7.05 ± 2.12 (n=19) | F=0.23 | <0.001 |
| Heterospecific Competitors | 1.24 ± 0.37 (n=42) | 0.51 ± 0.43 (n=34) | 0.84 ± 0.51 (n=19) | F=0.07 | 0.01 |
| Lewis’s Woodpeckers | 0.71 ± 0.25 (n=42) | 0 (n=34) | 0.58 ± 0.37 (n=19) | F=0.17 | <0.001 |
| European Starlings | 0.93 ± 0.35 (n=42) | 0.31 ± 0.18 (n=34) | 0.63 ± 0.51 (n=19) | F=0.05 | 0.02 |
| Surplus Cavities^a^ | 6.42 ± 1.69 (n=40) | 2.11 ± 0.98 (n=34) | 4.74 ± 1.92 (n=10) | F=0.0 | 0.26 |

^a^The number of suitable cavities minus the number of nesting competitors within a 50 m radius (0.785 ha) of each active nest.
